# A third-person perspective on co-speech action gestures in Parkinson's disease

**DOI:** 10.1016/j.cortex.2016.02.009

**Published:** 2016-05

**Authors:** Stacey Humphries, Judith Holler, Trevor J. Crawford, Elena Herrera, Ellen Poliakoff

**Affiliations:** a School of Psychological Sciences, University of Manchester, Manchester, United Kingdom; b Max Planck Institute for Psycholinguistics, Nijmegen, The Netherlands; c Department of Psychology, Centre for Aging Research, University of Lancaster, Lancaster, United Kingdom; d University of Oviedo, Oviedo, Spain

**Keywords:** Parkinson's disease, Gesture, Motor imagery, Language, Action simulation

## Abstract

A combination of impaired motor and cognitive function in Parkinson's disease (PD) can impact on language and communication, with patients exhibiting a particular difficulty processing action verbs. Co-speech gestures embody a link between action and language and contribute significantly to communication in healthy people. Here, we investigated how co-speech gestures depicting actions are affected in PD, in particular with respect to the visual perspective—or the viewpoint – they depict. Gestures are closely related to mental imagery and motor simulations, but people with PD may be impaired in the way they simulate actions from a first-person perspective and may compensate for this by relying more on third-person visual features. We analysed the action-depicting gestures produced by mild-moderate PD patients and age-matched controls on an action description task and examined the relationship between gesture viewpoint, action naming, and performance on an action observation task (weight judgement). Healthy controls produced the majority of their action gestures from a first-person perspective, whereas PD patients produced a greater proportion of gestures produced from a third-person perspective. We propose that this reflects a compensatory reliance on third-person visual features in the simulation of actions in PD. Performance was also impaired in action naming and weight judgement, although this was unrelated to gesture viewpoint. Our findings provide a more comprehensive understanding of how action-language impairments in PD impact on action communication, on the cognitive underpinnings of this impairment, as well as elucidating the role of action simulation in gesture production.

## 1 Introduction

Historically, Parkinson's disease (PD) has primarily been considered a movement disorder, characterised by cardinal motor symptoms such as tremor, rigidity, postural instability, and particularly slowness of movement. It is now well-recognised that PD leads to cognitive deficits in areas such as attention, memory, executive function and visuospatial abilities (Verbaan et al., 2007). This combination of motor and cognitive impairment can have a profound effect on language and communication, contributing significantly to reductions in quality of life for people with PD (Miller, Noble, Jones, & Burn, 2006). In addition to motor-based speech deficits which result in dysarthria and slowness of speech, PD patients show a reduction in performance on cognitive language tasks such as verbal fluency (Raskin, Sliwinski, & Borod, 1992), providing word definitions, interpreting metaphors, constructing sentences and naming objects (Lewis, Lapointe, Murdoch, & Chenery, 1998).

Over and above a more general difficulty with language, PD patients are particularly impaired on tasks where language has an action component. When asked to generate lists of verbs, patients perform significantly worse than on fluency tasks involving other semantic or phonological categories (Signorini & Volpato, 2006). While this deficit could reflect an impairment in executive function, because verbs are more cognitively complex than other parts of speech (Signorini & Volpato, 2006), accumulating evidence suggest it is attributable to the involvement of the motor system in representing verbs (see Cardona et al., 2013, for a review). First, Fernandino et al. (2013) found that PD patients were only impaired relative to controls when processing action verbs (e.g., to grasp, to squeeze), but not abstract verbs (e.g., to depend, to improve). This cannot be explained by executive dysfunction since abstract words are more cognitively complex than concrete words (Hoffman, Rogers, & Lambon Ralph, 2011). Second, it has been shown that PD patients make more errors when naming actions with a high motor content (e.g., digging) compared to a low motor content (e.g., sleeping) (Herrera, Rodríguez-Ferreiro, & Cuetos, 2012). Furthermore, impairments in action-verb production and action semantics have been found to occur in the early stages of the disease, in the absence of mild cognitive impairment, and are unrelated to executive function deficits (Bocanegra et al., 2015).

Despite the fact that impairments relating to action and language are well-documented in PD, one area which has received little attention in this clinical population is that of co-speech gestures, which embody a clear link between these two cognitive domains. That is, they are a form of action which is tightly linked to language (Willems & Hagoort, 2007). Co-speech gestures are the spontaneous movements of the hands and arms (and occasionally other body parts) which speakers frequently produce while talking (Kendon, 2004, McNeill, 1992). In healthy people, co-speech gestures are closely related to speech (temporally, semantically and pragmatically), and contribute a great deal to communication (Holler and Beattie, 2003, Hostetter, 2011). Several different types of co-speech gestures, with various functions, have been identified*. Iconic gestures* represent concrete semantic information, often depicting the size, shape, relative position or motion of an object, whereas *metaphoric gestures* represent abstract information (e.g., gesturing in higher gesture space to indicate high intelligence, McNeill, 1992). *Deictic gestures* are pointing gestures, where the speaker typically uses an extended finger or their hand to indicate a referent (Kendon, 2004, McNeill, 1992). Other gestures do not convey any semantic, propositional information themselves but play more of an interactive or pragmatic role. *Interactive gestures* refer to “some aspect of the process of conversing with another person” (Bavelas, Chovil, Lawrie, & Wade, 1992, p.473) such as checking understanding or regulating turn-taking, and *beats* are bi-phasic flicks of the hand which are used to add emphasis and beat the rhythm of speech (McNeill, 1992).

Some PD patients exhibit ideomotor apraxia when asked to pantomime movements (e.g., “use a hammer”), commonly making spatial errors such as using a body part to represent an object (Leiguarda et al., 1997). Pantomime gestures can be produced spontaneously during conversation, with or without words, especially when people describe action information. However, pantomimed movements produced during apraxia testing are different to the co-speech gestures we describe in this article, in that they are produced *on demand* in the absence of speech, whereas co-speech gestures are produced naturally and idiosyncratically as part of the speech-production process (Bernardis and Gentilucci, 2006, Kendon, 2004, McNeill, 1992, McNeill, 2008). Only a small number of studies have investigated how co-speech gestures specifically are affected in PD and their approach has been limited. An early study by Pitcairn, Clemie, Gray, and Pentland (1990) found that gesture production was significantly reduced in PD (*N* = 4), but they analysed gestures without considering the concurrent speech and did not consider all types of gesture. These shortcomings were addressed by Cleary, Poliakoff, Galpin, Dick, and Holler (2011) who found no difference between PD patients and controls in gesture rate per 100 words of speech (which takes into account the slower rate of speech in PD) and no difference in terms of the percentage of gestures of each type produced (iconic, metaphoric, deictic, pragmatic and interactive). Participants were video-recorded whilst they described simple actions (pressing a button and turning a door handle) that they had actually performed during an earlier experiment. Whilst gesture rate was unimpaired, the gestures of the PD patients were significantly less precise than controls (e.g., using their whole hand with a downward movement to indicate pressing a button, rather than extending and moving down a single finger), despite the fact they were capable of performing the actions earlier, and that their gesture precision was unrelated to motor symptom severity. One possible explanation is that the simplification of the gestures reflects a reduction in spontaneous motor expressivity not captured by standard motor symptoms scales. Alternatively, as gestures are thought to rely on underlying mental imagery (Feyereisen & Havard, 1999), this study may suggest that the cognitive representation of the actions underlying the gestures may be less detailed, less accessible, or otherwise affected in PD.

The latter interpretation is consistent with the Gesture as Simulated Action framework (Hostetter & Alibali, 2008) which proposes that gestures arise from motor simulations which underlie cognitive representations and imagery. When a gesture represents an action we can assume this is based on an underlying cognitive representation of that action, which should activate motor imagery of either an explicit (where participants are specifically asked to imagine a movement) or implicit (where imagery is not directly instructed) nature. Both types of motor imagery have been shown to be slowed in Parkinson's, such as when people are asked to imagine a sequential finger movement (explicit) or judge the laterality of rotated images of hands (implicit) (Dominey, Decety, Broussolle, Chazot, & Jeannerod, 1995). If the production of action gestures does indeed rely on action representations involving simulations of motor movements, it is reasonable to expect PD patients to have difficulty producing these kinds of gestures.

One aspect of gesture which provides a window into how the gesturally-depicted action is cognitively represented is the viewpoint (or visual perspective) that the speaker takes when producing the gesture. McNeill (1992) describes character viewpoint (C-VPT) gestures, where the speaker takes on the role of the person enacting the action, from a first-person perspective, placing their own body in the event. In C-VPT gestures the speaker would use their own hands to represent the hands of the character they are describing, by “acting out” the action. For example, a gesture describing the actions of a skier involving clenching the hands into fists while moving them synchronously up and down at the sides of the body to depict someone using skiing poles would be classed as C-VPT. Conversely, observer viewpoint (O-VPT) gestures occur from a third-person perspective. In this case, the speaker's body remains external to the event; rather than the hands representing the character's hands, the speaker's hand represents the character (and his/her actions) as a whole. A gesture produced using the index finger to trace the slalom skiing path a skier followed down a hillside would be classed as O-VPT.

On the one hand, it might be predicted that PD patients would produce low levels of O-VPT gestures. It has been demonstrated that speakers with higher fluid intelligence produce more gestures from non-egocentric (O-VPT) views than speakers with average fluid intelligence on a task involving visual imagery (Sassenberg, Foth, Wartenburger, & van der Meer, 2011). Because PD causes mild cognitive impairment in most patients, PD patients might produce fewer O-VPT gestures than controls, who would likely have higher fluid intelligence. One hypothesis suggests that O-VPT gestures are more sophisticated than C-VPT gestures as they reflect the ability to form abstract and flexible mental representations (Sassenberg et al., 2011). Alternatively, it has been suggested that C-VPT gestures may in fact be more cognitively complex than O-VPT gestures because they depend on the ability to take another's perspective (Cartmill, Beilock, & Goldin-Meadow, 2012) which involves executive function. This might suggest that PD patients would find CVPT gestures more difficult to produce due to their frontostriatal cognitive degeneration (Owen, 2004). Regardless of which viewpoint is more complex, the Gesture as Simulated Action framework predicts increased production of C-VPT gestures when the underlying mental representation involves motor imagery, and increased production of O-VPT gestures when the underlying mental representation involves visual imagery (Hostetter & Alibali, 2008).

Given PD patients' specific impairment in action semantics, we were interested in exploring gesture production in a task which relies more on motor imagery than visual imagery to assess whether action gestures are also affected in line with action verb processing. There is some evidence that people with Parkinson's favour a third-person strategy during motor imagery. When mentally rotating hands, patients use the same brain areas normally activated in healthy people (the posterior parietal–dorsal premotor circuitry), whilst also showing additional activation in the occipito–parietal cortex and left extrastriate body area (EBA) (Helmich, de Lange, Bloem, & Toni, 2007). This suggests that PD patients may compensate for their impaired motor system by using a third-person viewpoint or visual imagery strategy (Helmich et al., 2007). If the ability to imagine and represent movement from a first-person perspective is impaired in PD, this may affect how first- and third-person viewpoints are used in gesture.

The present study therefore aimed to further investigate the nature of the mental representations underlying iconic action gestures in PD patients. Speakers' gestures were analysed as they described viewed actions, whilst the following research questions were considered. (1) Does Parkinson's affect the rate at which gestures are produced, and is this different for different kinds of gestures? Cleary et al.'s (2011) study suggests no difference in the rate of gesture production whilst describing a limited range of actions. We sought to replicate and extend this finding by looking at a wider range of everyday actions. We also made the task communicative by asking participants to describe the information to an addressee other than the experimenter, who they were told was unfamiliar with the material. (2) Does Parkinson's affect the viewpoint employed in gesture when talking about actions? Since people with Parkinson's may rely on third-person visual compensatory strategies when doing tasks involving motor simulation or imagery, we predicted that people with Parkinson's would produce more gestures from a third-person, O-VPT than healthy controls. Gesture viewpoint is likely to be less influenced by physical motor impairment than gesture precision (Cleary et al., 2011) as C-VPT and O-VPT gestures should still be easily recognisable even when they are less precise. Thus, examining gesture viewpoint allows for a clearer investigation of whether cognitive representations of action affect gesture production in PD. (3) Is action naming and motor imagery ability in Parkinson's related to the rate of gesture production and/or the viewpoint employed in gesture? We used a perceptual weight judgement task (Poliakoff, Galpin, Dick, & Tipper, 2010) as a proxy measure of motor imagery ability, and hypothesised that performance on this task would be related to gesture viewpoint. We also used a new version of the action naming task employed by Herrera et al. (2012), in which we asked participants to name not only static pictures of actions, but also dynamic video clips of actions. Participants completed this task in part to provide an initial exposure to the actions for the later description task, so they had already had the opportunity to name each of the actions that they later described. Our analyses here were more exploratory, and were motivated by the idea that action naming and action gesture production may both rely on the same cognitive representations of action.

## 2 Materials and methods

### 2.1 Participants

29 people with Parkinson's and 28 age-matched controls participated in the study, which was approved by the local NHS research ethics committee (reference 11/NW/0143). Patients who had received a clinical diagnosis of idiopathic PD were recruited via referral from a consultant neurologist at a local Parkinson's clinic, or via advertisements placed in the Parkinson's UK magazine and website. Some of the age-matched controls were spouses or friends of the Parkinson's participants. The remainder of the controls were recruited via advertisements at the University of Manchester and community groups.

Participants were excluded if they had an auditory or visual impairment rendering them incapable of understanding verbal instructions or viewing images on a computer screen, a neurological disease other than PD, if they scored outside the normal range on the Mini-Mental State Examination (Folstein, Folstein, & McHugh, 1975) for dementia screening (<25/30), or if they had previously suffered a serious head injury or a stroke. All participants were native speakers of English.

Two Parkinson's patients were excluded from the study entirely because of severe dyskinesias (involuntary movements as a side effect of medication) which impaired their performance on all tasks and made gesture analysis impossible. Two further Parkinson's patients were excluded from the gesture-specific analyses because they misunderstood the communicative task (mistakenly believing that they were not allowed to mention the name of the action they were asked to describe, leading to the production of contrived, pantomimed actions as opposed to spontaneous co-speech gestures). Three controls were also excluded from the gesture-specific analyses. One person was unwilling to be video-recorded and two had missing video data because of technical errors. In total, 27 PD patients and 28 controls were included in the non-gesture analyses, and 25 PD patients and 25 controls were included in the gesture analyses.

All participants completed a battery of neuropsychological tests (see Table 1), including the Stroop test of executive function (Stroop, 1935), digit span forwards and backwards (Wechsler, 1997), the National Adult Reading Test as a measure of pre-morbid IQ (Nelson, 1982), the Mill Hill vocabulary scale as a measure of reproductive verbal intelligence (Raven, Raven, & Court, 1988), the Geriatric Depression Scale (Yesavage & Sheikh, 1986) and verbal fluency tasks (Lezak, 2004). The PD patients exhibited significantly higher levels of depression, and significantly lower verbal IQ than the control group. One PD patient was left handed, as was one control.

The Parkinson's patients completed the session on their normal medication, at a time of day selected by the participant to maximise a stable “on” period. Motor symptom severity was rated using the motor subsection of the Unified Parkinson's Disease Rating Scale (UPDRS) (Fahn & Elton, 1987). All patients were recruited at Hoehn and Yahr stage III or less (Hoehn & Yahr, 1967). The majority (21) of patients were at stage 2 indicating bilateral motor symptoms without impairment of balance. Five patients were at stage 1, and one was at stage 3. All but two of the Parkinson's patients were taking dopaminergic medication and on/off fluctuations were assessed using the UPDRS (maximum fluctuation score = 7). The majority (17) of the patients reported no on/off fluctuations, with the remaining 8 patients reporting varying degrees of fluctuations with scores of 2–4. See Table 2 for the clinical features of the Parkinson's group.

### 2.2 Procedure

After completing the battery of neuropsychological tests outlined above, participants completed an action naming task. Participants viewed 40 photographs and 40 short video clips which depicted every day actions and were required to name the actions as quickly as possible. The video stimuli were collected from the Verb and Noun (VAN) test (Webster & Bird, 2000), whereas the photographs were copyright-free images found using google images. The verbs denoting the actions depicted in the stimuli in each condition (picture *vs* video) were matched on verb frequency, age of acquisition and imageability. The final stimulus set was made up of pictures and video clips which depicted verbs that were rated by 14 undergraduate students (see Herrera et al., 2012) for the amount of movement required to perform the action on a 1–7 Likert scale, where 1 represented “no movement” and 7 represented “full movement”. Subsets of 20 “high” (>5) motion-content actions (e.g., skiing) and “low” (<3) motion-content actions (e.g., sleeping) were then selected for each stimulus type (pictures and videos). The stimuli were presented for four seconds each, using Presentation^®^ software (Version 0.70, www.neurobs.com), and participants responded vocally via a microphone.

Participants then completed a perceptual weight judgement task (for details see Poliakoff et al., 2010), again using Presentation^®^ software, where they viewed short video clips of a person's hand moving to pick up a plastic box from a table and place it on a higher surface, and were asked to guess the weight of the box on a 9-point scale from 50 g to 450 g in increments of 50. The same box was seen in all the clips, but it varied in weight (either 100 g, 200 g, 300 g or 400 g), so the weight information could only be gleaned from the movement parameters of the actor.

Finally, participants were video-recorded while they completed an action-description task. A randomly selected subset of 10 photographs and 10 video clips of actions that they had previously seen during the naming task were presented again, and participants were asked to describe the stimuli in as much detail as possible to a confederate addressee. The stimuli were presented in a randomised order and participants viewed all the photographs or all the videos first, counterbalanced across participants. The photograph or the final frame of the video remained onscreen throughout their description; however, the screen was positioned at the side of the participant to facilitate engagement with the addressee and to allow any gestures to be recorded and visible to the addressee. To encourage rich descriptions and to make the situation appear communicative, the participant was told that the addressee was trying to match their description to a separate set of stimuli showing the correct option as well as a selection of similar alternatives. Two addressees were used in total (only one per participant). In order to ensure some degree of consistency across participants, the addressees did not talk back to the participant during their explanation (participants were told the addressee was not allowed to ask questions) but indicated their engagement and understanding through eye contact and backchannel responses (such as nodding and “mm-hmm” vocalisations, Yngve, 1970). Participants were fully aware that they were being video-recorded and that their communication would be evaluated, but they were unaware that the focus of the study was on gesture.

### 2.3 Analysis

#### 2.3.1 Action naming

We recorded participants' vocal responses and examined both accuracy and reaction times. The sound files were imported into the phonetic software Praat (http://www.praat.org; Boersma & Weenink, 2015) so that we could accurately establish the onset of the vocal response using the beginning of the waveform of the sound. Responses were time-locked to the onset of the photograph or video-clip. We scored closely synonymous verbs as correct (e.g., crouching or squatting). However, only the participant's first response was scored, even if they later self-corrected, so as not to confound reaction times. To take into account the fact that the video clips varied in terms of when the action became clear (e.g., some videos began with the action mid-flow whereas in others it took a second or two for the action to begin) 11 younger controls (mean age: 28) completed both the picture and video action naming tasks to establish baseline mean “minimum” RTs. We then subtracted these mean RTs for each stimulus from the individual RTs generated by the actual research participants to provide baseline-corrected RTs.

#### 2.3.2 Gesture coding

The participant video recordings were imported into the software ELAN (http://tla.mpi.nl/tools/tla-tools/elan/; Sloetjes & Wittenburg, 2008) for the identification of all co-speech gestures. Unless they occur in direct sequence, gestures are usually triphasic and consist of preparation, stroke (the most meaningful component of the gestural movement) and retraction (McNeill, 1992). Each stroke phase was therefore considered as constituting one gesture. In cases where multiple gestures were produced in succession without the hands returning to rest, each separate gesture stroke was identified as a new gesture and annotated accordingly. Any non-communicative self-grooming movements were not coded (such as rubbing or scratching the face). A second coder, who was blind to the experimental hypotheses and to the group status of the participants, independently identified all gestures produced within the first 25% of the total time that each participant spoke for (562 gestures in total) for the purposes of establishing inter-rater reliability. This period was selected because the main coder found that the first 25% of the descriptions were the most gesture-rich, perhaps indicating that the elderly participants became more fatigued as time went on and produced fewer gestures towards the end. The procedure resulted in 90.75% agreement for gesture identification.

The speech produced by the participants during their descriptions was transcribed verbatim. Any speech and gestures produced that were not part of the action-descriptions (such as when asking for clarification of the task) were excluded. The total number of words used and the total number of gestures produced were counted for each participant and used to calculate the number of gestures produced per 100 words of speech for each participant, to take into account speech rate.

All gestures were classified according to four gesture types outlined in the Introduction – iconic, metaphoric and deictic, with interactive gestures and beats collapsed into one final category summarising gestures with a pragmatic function (Kendon, 2004). Gesture coders saw the stimuli prior to coding. Gestures were coded with reference to the verbal statements accompanying them rather than without audio, to help clarify the underlying concept that the gesture referred to (cf. Feyereisen and Havard, 1999, Trafton et al., 2006). This facilitated the identification of action gestures which were not always immediately obvious from the gesture form alone. For example, gesturing with a pointed finger flicking up and down might refer to a man jumping up and down, or it might refer to the vertical pipe on the wall behind him. Being able to identify gestures which communicated action information was critical to our analysis and would not have been possible without reference to the verbal statements. For each participant, we calculated the proportion of each type of gestures that they produced out of their individual total. A second, independent coder who was blind to the experimental hypotheses then re-coded 25% of the gestures from each participant according to gesture type, i.e., iconic, metaphoric, deictic and interactive gestures (399 gestures in total). This resulted in an overall percentage agreement of 92.98% and a Cohen's Kappa = .71, indicating a high level of agreement (Landis & Koch, 1977). Evaluating the reliability of each gesture type individually revealed 92.24% agreement and Cohen's Kappa .75 for classifying gestures as iconic, 96.24% agreement and Cohen's Kappa .71 for classifying gestures as deictic, and 96.24% agreement and Cohen's Kappa .66 for classifying gestures as interactive, all indicating good to high levels of agreement (Landis & Koch, 1977). The reliability analysis for metaphoric gestures revealed 99.25% agreement and Cohen's Kappa −.003. This high % agreement but low kappa is a common paradox produced when many cells in the table are 0 or <5 (see Cicchetti & Feinstein, 1990) which arose because there were so few metaphoric gestures overall in this dataset (*n* = 11). For this reason, the Kappa value cannot be meaningfully interpreted for this gesture type.

For the purpose of the gesture viewpoint analysis, only iconic gestures were considered as they are the only gesture type that can demonstrate viewpoint in the context of action depiction. Iconic gestures were first classified as to whether or not they depicted action information. A second, independent coder who was blind to the experimental hypotheses re-coded 25% of the iconic gestures from each participant for action content (294 gestures in total) resulting in a percentage agreement of 97.28% and a Cohen's Kappa = .92, indicating very high agreement (Landis & Koch, 1977).

Iconic action gestures were classified as either C-VPT or O-VPT. As in Parrill (2011), gestures were classified as C-VPT if the speaker's hands mapped directly onto the character's hands they were describing. Gestures were classified as O-VPT if the hands represented an entire body or object as though describing the scene from a third person perspective (see Introduction for examples of C-VPT and O-VPT “skiing” gestures found in this dataset). Having previously viewed the stimuli, gesture coders were able to make these classifications accurately. For each participant, the proportion of their total viewpoint gestures which were classed as C-VPT was calculated (with O-VPT gestures constituting the complement of this). A second, independent coder who was blind to the experimental hypotheses re-coded 25% of the iconic action-gestures from each participant for viewpoint, resulting in a percentage agreement of 92.74% and a Cohen's Kappa = .88, indicating high agreement (Landis & Koch, 1977).

It is worth mentioning a special class of C-VPT gestures known as “body as reference point” (BARP) gestures identified by Holler and Beattie (2002). BARP gestures involve the speaker referring to their own body when describing the body of another, but without the speaker's hands mimicking the character's hands. For example, the speaker may describe the character's beard by “drawing” it onto their own face, or may describe the length of a character's hair by touching their own head and moving the hands downward. We found many examples of BARP gestures in our dataset, but we did not include them as C-VPT gestures as they were not representing the character's action, but the character's appearance. However, we re-ran our analysis with BARP gestures included as C-VPT gestures and the results did not change.

#### 2.3.3 Statistical analysis

To examine gesture rate, we calculated the number of gestures produced per 100 words of speech and compared this between groups using an independent samples *t*-test. To analyse differences in the production of the four gesture types and the two gesture viewpoints, subtypes within each set of categories were first converted to percentages to take into account individual variations in overall gesture rate. The percentages for each gesture type were then compared between groups using independent samples *t*-tests.

Our main analysis of interest concerned gesture viewpoint. Given that depression (GDS) and verbal intelligence (NART) differed significantly between the two groups, these factors were controlled for in the subsequent gesture viewpoint analysis. We included both factors as covariates in a univariate ANOVA comparing the percentage of C-VPT gestures produced between the two groups.

Multiple regression analyses were then conducted to examine whether mean action naming speed or weight judgement ability (summarised by the R^2^ value of linear regression between the actual weights and the participants' judgements) predicted either overall gesture rate or the proportion of C-VPT gestures produced.

## 3 Results

### 3.1 Action naming task

Controls responded significantly faster than patients in high-motion conditions, but not in low-motion conditions, although the group difference for the video low-motion condition demonstrates a trend towards significance (Table 3).

### 3.2 Weight judgement task

**Fig. 1:**
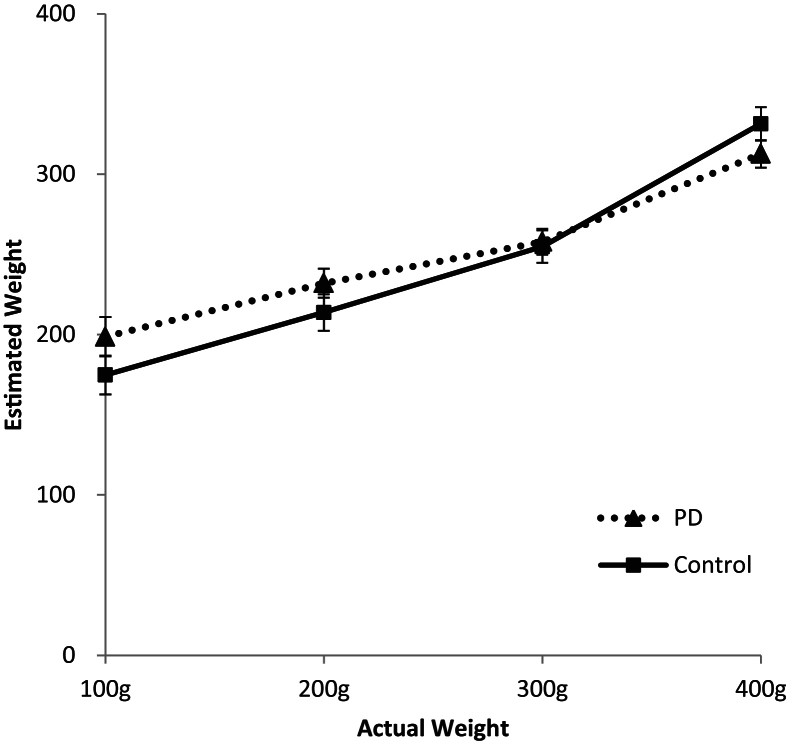
Mean weight estimates (in grams) compared to the actual weights (in grams) of the blocks for PD patients and controls.

To analyse performance on the weight judgement task, each participant's mean response to each weight level was calculated (see Fig. 1 above). Both groups were able to do the task in that their weight judgements increased as did the weights themselves. However, both groups showed a tendency to overestimate the lighter weights and underestimate the heavier weights, leading to a narrower range of estimates than the actual range of weights and suggesting that they found the task difficult. Performance on the task for each participant was summarised by the R^2^ value of the linear regression between the actual weights and the participant's judgements and Fig. 1 illustrates that the slope was steeper for the control than the PD group, suggesting that their performance was more accurate. This was confirmed statistically with a significantly lower R^2^ value for the PD patients than controls (see Table 3).

### 3.3 Gesture rate and gesture types

In total, 1440 gestures were identified and coded in ELAN. Twenty-five PD patients and 25 controls were included in the gesture rate analysis. Five PD patients and two controls did not produce any gestures. Though not analysed statistically, observation of the videos showed that in addition to not gesturing, these five PD patients performed no self-grooming movements at all whereas the two controls did. There were no group differences in the rate of gesture production per 100 words (see Table 3). In the PD group, the rate of gesture production was not correlated with the level of motor symptom severity as assessed by the UPDRS (*r* = −.104, *p* = .61).

We asked whether gesture rate when describing actions could be predicted by performance on tasks involving action representation. A multiple regression was performed to assess whether group (PD patient or control), performance on the weight judgement task or mean action naming speed (static actions only, baseline corrected) predicted gesture rate, however the overall model was not significant [*R*^2^ = .073, *F*(3, 48) = .612, *p* = .657]. Evaluating each predictor individually also did not reveal any significant effects.

In addition, we did not find any group differences in the proportion of gestures classified as iconic, metaphoric, deictic or interactive/pragmatic (see Table 3). Overall, the pattern of gesture type usage was very similar between the groups.

### 3.4 Gesture viewpoint

**Fig. 2:**
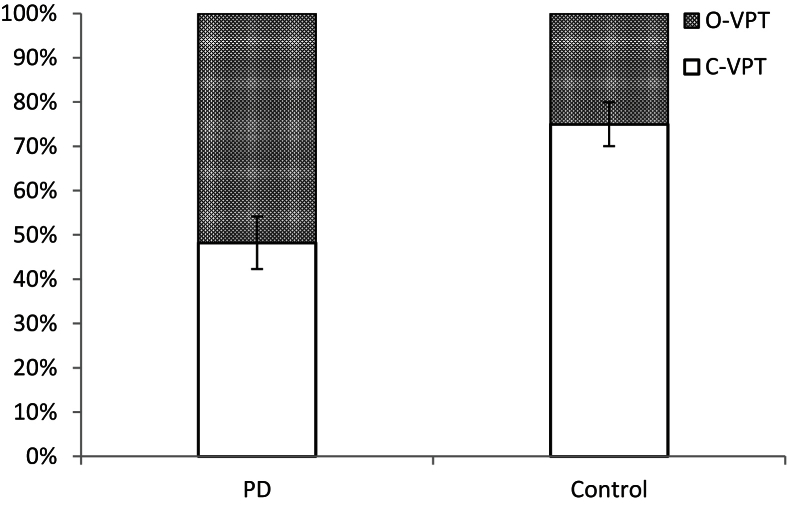
Mean proportions of C-VPT and O-VPT gestures for PD patients and controls.

Eighteen PD patients and 22 controls were included in this analysis, as the remainder did not produce any iconic action-gestures depicting viewpoint. In total, 491 viewpoint gestures were analysed (see Table 4). For each participant, the percentage of iconic action-gestures categorised as observer and character viewpoint was calculated. An independent samples *t*-test revealed a significant group difference [*t*(38) = 3.395, *p* = .001], with controls producing proportionally more C-VPT gestures (mean = 74.99%, SD = 23.28) than PD patients (mean = 48.21%, SD = 25.11) (see Fig. 2). The significant effect of group on proportion of C-VPT gestures remained after controlling for depression and verbal intelligence [*F*(3, 36) = 5.702, *p* = .003]. Finally, there was no relationship between motor-UPDRS score and the proportion of C-VPT gestures in the PD patient group (*r* = −.3, *p* = .27).

A multiple regression was conducted to assess whether group (PD or control), performance on the weight judgement task or mean action naming speed predicted the proportion of C-VPT gestures, and whilst the overall model was significant [*R*^2^ = .27, *F*(3, 36) = 4.34, *p* = .01], only group was a significant predictor (*β* = .537, *p* = .001). Weight judgement performance (*β* = .036, *p* = .81) and action naming speed (*β* = .163, *p* = .3) did not predict gesture viewpoint.

## 4 Discussion

The present study aimed to elucidate how changes in action-representation might affect gesture production in PD. We examined performance in tasks thought to engage these processes (weight judgement and action naming) and explored the manner in which action information is expressed in gesture in people with PD and healthy age-matched controls.

In accordance with Cleary et al. (2011), no difference in the rate of gesture production per 100 words spoken was found between the Parkinson's patients and the controls. Furthermore, gesture rate was not correlated with motor symptom severity in the Parkinson's group. This suggests that there is not a straightforward reduction in gesture use in early PD, despite the fact that movement generally is slowed and reduced in these participants. This finding shows that gesture use is intrinsic to communication and speech production, even in a movement-impaired sample. However, it is likely that reductions in gesture production would be seen in patients with more severe motor symptoms. That said, 20% of the PD group did not gesture at all and did not produce any self-grooming movements, compared to 8% of the control group who did not gesture but still produced self-grooming movements. This suggests an overall reduction in hand movements in some mild-moderate PD patients. One explanation could be embarrassment related to tremor in PD which has been suggested to lead to the avoidance of hand movements during speaking (Cleary et al., 2011). Although we did not find a relationship between gesture rate and motor UPDRS score, the lack of gesture use in some patients may reflect more subtle motor impairment or psychosocial changes involving embarrassment and anxiety when interacting with others.

Whilst there was no quantitative change in gesture production in the Parkinson's patients, the results suggest that PD can influence qualitative aspects of gesture production when describing actions. We found that healthy older adults tended to produce more gestures from a character-viewpoint when describing actions, whereas people with Parkinson's produced more gestures from an observer-viewpoint, and that this difference cannot be explained by higher levels of depression and lower verbal intelligence in Parkinson's patients. This suggests that the way actions are cognitively represented may have changed in PD, and complements previous work demonstrating the effect of Parkinson's on verbal language with an action component (Fernandino et al., 2013, Herrera et al., 2012, Signorini and Volpato, 2006).

One possible explanation is that PD patients are less able to cognitively simulate the action that they are asked to describe. As described in the introduction, when PD patients mentally rotate hands, the EBA shows significant activation which is absent in controls (Helmich et al., 2007). The EBA is involved viewing body parts. It responds more to static aspects of the human form rather than dynamic motion (Downing, Peelen, Wiggett, & Tew, 2006), and, critically, to allocentric (third person) views of bodies more than egocentric (first person) views (Chan et al., 2004, Saxe et al., 2006). A study using continuous theta-burst stimulation (cTBS) to interrupt brain function suggested that the EBA may be compensating for a function normally performed by the dorsal premotor cortex. Typically, mental rotation of viewed hands improves when they match the posture of the participant's own hand. However, when the EBA was inhibited with cTBS, the benefit of this posture congruency effect was lost in PD patients but not in controls. Conversely, cTBS of the dorsal premotor cortex reduced performance in the control group, but not the PD group (van Nuenen et al., 2012). This compensatory effect during motor imagery in PD may therefore underlie the gesture viewpoint finding reported in the present study. PD patients may be less able to imagine or cognitively simulate the actions from a first person perspective, and so rely more on third-person, visual information to represent the action, which then influences the viewpoint of the subsequent gesture.

From a theoretical perspective, our results support the notion that action gesture production in healthy people relies on motor-based action representations, in line with the Gesture as Simulated Action framework (Hostetter & Alibali, 2008). The GSA framework also predicts that gestures produced as a result of motor imagery are more likely to be C-VPT gestures, whereas gestures produced as a result of visual imagery are more likely to be O-VPT gestures. We propose that the viewpoint findings reported in this study reflect a reliance on, or preference for, visual imagery over motor imagery when representing or simulating actions in PD. Taken together, our findings corroborate the notion that while simulations of motor movements and visual imagery may underlie gestural actions, as predicted by the GSA framework, they also appear to be connected with the linguistic system in a way special way compared to goal-directed motor movements (Cole, Gallagher & McNeill, 2002). This may account for the preserved gesture rate in PD patients despite motor impairments.

In relation to other measures of action representation, we did not find any relationship between action naming speed or performance on the weight judgement task and gesture rate or viewpoint. We hypothesised that if performance on the weight judgement task reflects motor imagery ability, that this should be related to the ability to produce action gestures from a first person perspective. However, although we did find an overall group difference in performance on this task, PD patients are still able to do the task to a degree, that is, their weight estimates do increase in line with the increase in actual weight (Poliakoff et al., 2010). Therefore, it is possible that patients are able to do the task by relying more on visual information than kinematics (cf. Helmich et al., 2007). Indeed, it has previously been shown that even healthy participants rely on a mixture of visual and kinematic cues to perform this task (Hamilton, Joyce, Flanagan, Frith, & Wolpert, 2007), which could account for why we did not find a relationship between weight judgement performance and gesture viewpoint.

We also replicated the finding of Herrera et al. (2012), that PD patients were significantly slower than controls to name actions with a high motor component (either in still or dynamic form), but not when naming actions with a low motor component. This is consistent with a difficulty in simulation, but we did not find a relationship between naming speed and gesture viewpoint. Speed was not a factor during the action-description task, however, as participants were given unlimited time to describe the actions in as much detail as they could. The fact that we did not find a relationship may be because the naming task reflects only the speed with which patients can simulate actions, whereas the viewpoint finding reflects the quality of the simulation itself. Hickok (2010) argues against the involvement of the motor system in action semantics, stating that motor information may contribute to but is not necessary for the understanding of action information. After all, with an impaired motor system in PD it is not that people can no longer understand action concepts, but that their ability to access action representations is slowed down. Similarly, although we found that the production of C-VPT gestures was significantly reduced in PD, the patients in this study did still produce some gestures from a first person perspective. This could indicate that because of their impaired motor system, simulating others' actions from a first person perspective may be more demanding in PD but is certainly not impossible. Indeed, two patients who were excluded from the statistical analyses for mistakenly believing they were not allowed to mention the name of the action during their description relied more on first person pantomimes as a way of communicating the information they thought they were not allowed to verbalise. This might suggest that when the demands of the situation require it, PD patients can produce gestures from a first person perspective, but in normal conversation the third person perspective may have become an easier way of simulating others' actions and thus preferred. The viewpoint findings from the present study are interesting because they suggest that whilst the involvement of the motor system may not be critical for the understanding of action, it can still influence how people are able to communicate and share information about actions.

A potential limitation of this study is that apraxia was not assessed. Whilst more commonly associated with atypical parkinsonism, some PD patients exhibit mild ideomotor apraxia beyond extrapyramidal deficits of the disease, as described in the introduction (Leiguarda et al., 1997). Whilst the relationship between apraxia and co-speech gesture has rarely been explored in the literature, it is possible that apraxia could influence gesture production in PD. We suggest an influence here is unlikely for several reasons. Ideomotor apraxia appears to be associated with disease severity, being close to absent in Hoehn & Yahr stage 1 and reaching nearly 40% at stage 4 (Vanbellingen et al., 2012). With the majority of patients in this study at stage 2, we would expect apraxia to be present in only a small number of our participants. As we did not find a difference in gesture rate between the two groups, it would appear unlikely that apraxia could have had a significant influence on gesture production in this study. Furthermore, evidence from split-brain patients who exhibit unilateral apraxia yet nonetheless strongly prefer to gesture communicatively with the apraxic hand suggests that the production of co-speech gestures relies on processes other than those subserving praxis (Lausberg, Zaidel, Cruz, & Ptito, 2007). In addition to gesture production per se, apraxia could be hypothesised to affect the form of gestural depictions, for example by not situating the movement in the correct gesture space (external configuration error), or by failing to leave space in the gesture for an imaginary tool (internal configuration error). However, a gesture could still be classed as C-VPT despite having a configuration which would normally be considered an error in a pantomime task.^1^

A second potential criticism which could be levelled at this study, is that the gesture findings may not reflect a change in action-representation as we propose, but may instead simply reflect the fact that some types of movements are easier to perform than others, making them more preferred movements for PD patients. This account is unlikely since the extent of movement required to perform either a C-VPT or O-VPT gesture did not appear to favour O-VPT gestures in terms of simplicity. For example, a C-VPT skiing gesture could be achieved by simply placing both hands roughly at the sides of the body with a loosely closed fist, whereas O-VPT skiing gestures seen in this study involved one hand being brought up high in front of the face with a pointed finger and moving down in an S-shape towards the abdomen. Secondly, there was no relationship between general motor symptom severity and any of our gesture outcome measures, suggesting that impaired motor function alone cannot account for changes in gesture viewpoint. Overall, our results can be explained by a reliance on third person visual action information, consistent with existing literature on action representation and motor imagery in PD.

This is the first comprehensive analysis of gestural action communication in Parkinson's, in which we have demonstrated that PD reduces the production of action-gestures produced from a first person perspective. We propose that this finding is related to a difficulty in simulating actions from a first person perspective and a reliance on third person, visual features. Our examination of action-gesture production in Parkinson's provides a window into the cognitive processes underlying action representation in PD, as well as the processes underlying action gesture production in healthy participants.

## Figures and Tables

**Table 1:** Mean (SD) demographic characteristics and neuropsychological assessment of the Parkinson's (PD) and age-matched controls groups.

	PD patients	Controls	*t*	*p*
Gender	19 M, 8 F	12 M, 16 F		
Age	63.38 (6.59)	64.34 (5.65)	.581	.56
Education	14.59 (3.53)	16.17 (3.13)	1.785	.08
Geriatric Depression Scale	3.34 (2.66)	1.03 (1.22)	**4.3**	<.001*
Digit span forwards	6.97 (1.02)	6.86 (.98)	.386	.701
Digit span backwards	4.79 (1.44)	4.97 (1.01)	.513	.61
Mill Hill vocabulary	22.14 (4.3)	25.1 (3.57)	**2.936**	.005*
Verbal IQ (NART)	115.07 (7.93)	119.81 (5.29)	**2.535**	.014*
Stroop interference	.19 (11.12)	−1.06 (9.16)	.096	.924
Phonetic fluency	17.33 (6.99)	18.08 (3.89)	.474	.638
Semantic fluency	25.62 (5.99)	27.9 (4.51)	1.555	.126

* Indicates significant group differences.

**Table 2:** Clinical features of Parkinson's group.

	Mean	SD
Age of Onset	57.48	6.92
Disease duration	6.28 years	3.47
Levodopa equivalent dose	568.6	302.42
Motor UPDRS	22.44	8.89
Hoehn and Yahr staging (1–4)	1.85	.46
Motor fluctuations (0–7)	1.04	1.65
Laterality	14 L, 11 R^a^	

a 2 patients were unsure of side of onset, with both sides now equally affected.

**Table 3:** Mean (SD) baseline-corrected reaction times for action-naming (in msec), weight judgement task performance summarised by R-squared, gesture rate per 100 words and the proportion of gestures classified as each different type for PD patients and controls.

	PD patients		Controls		*t*	*p*	*d*	95% C.I.
Mean	SD	Mean	SD
Pictures-high motion	381.26	301.58	235.83	243.05	**2.01**	.05*	.53	.29 to 290.57
Pictures-low motion	402.14	271.73	296.68	250.36	1.53	.133	.40	−33.16 to 244.08
Videos-high motion	380.86	311.76	216.5	222.12	**2.3**	.025*	.61	21.07 to 307.65
Videos-low motion	432.49	284.37	298.73	237.03	1.93	.06	.51	−5.01 to 272.52
Weight judgement R^2^	.16	.15	.27	.15	**2.74**	.008*	.75	−.19 to .03
Gestures per 100 words	1.7	1.92	2.17	3.05	.54	.595	.19	−2.28 to 1.38
% Iconic	69.48%	.27	72.48%	.22	.41	.684	.12	−.18 to .19
% Metaphoric	.08%	.01	1.23%	.03	1.65	.106	.5	−.03 to .00
% Deictic	10.51%	.13	10.91%	.13	.10	.919	.08	−.07 to .08
% Interactive	18.5%	.19	15.38%	.2	.53	.599	.16	−.09 to .15

* Significant group differences.

**Table 4:** Total number of character viewpoint and observer viewpoint gestures produced by each group.

	PD patients	Controls
C-VPT	85	223
O-VPT	112	71

## Footnotes

1 If a participant produced a C-VPT smoking gesture, we did not require the hand to be brought right up to the lips (external configuration apraxia error) in order to count it as a C-VPT gesture as long as the gesture was still easily recognisable as a first-person smoking action. Similarly, when describing pulling, a participant would not be “penalised” for producing completely closed fists as part of the pulling gesture rather than leaving space for the imaginary rope (internal configuration apraxia error) in order for this to be counted as a C-VPT gesture.
